# Clinical Features and Prognosis of Cervical Esophageal Cancer

**DOI:** 10.3390/jcm14113803

**Published:** 2025-05-29

**Authors:** Dae-Gon Ryu, Keekyoung Kim, Hongqun Liu, Samuel S. Lee, Sangjune Laurence Lee

**Affiliations:** 1 Department of Internal Medicine, Pusan National University School of Medicine and Research Institute for Convergence of Biomedical Science and Technology, Pusan National University Yangsan Hospital, Yangsan 50612, Republic of Korea; gon22gon@naver.com; 2 Department of Biomedical Engineering, Schulich School of Engineering, University of Calgary, Calgary, AB T2N 4N1, Canada; keekyoung@gmail.com; 3 Liver Unit, University of Calgary Cumming School of Medicine, Calgary, AB T2N 4N1, Canada; hliu@ucalgary.ca (H.L.); samlee@ucalgary.ca (S.S.L.); 4 Division of Radiation Oncology, University of Calgary, Tom Baker Cancer Centre 1331 29 Street NW, Calgary, AB T2N 4N2, Canada

**Keywords:** esophageal cancer, squamous cell carcinoma, chemoradiotherapy

## Abstract

**Background:** This study aimed to analyze the clinical features and prognosis of cervical esophageal squamous cell carcinoma (ESCC) compared to ESCC more inferiorly in the esophagus. **Methods:** Medical records of patients with ESCC between December 2008 and December 2024 were retrospectively reviewed. A total of 497 patients with ESCC were included and divided into cervical (n = 32) and non-cervical (n = 465) groups. Clinical features and survival according to treatment were compared between the two groups. **Results:** Patients with cervical ESCC were older (median age 73 years vs. 67 years, *p* = 0.047) with a higher proportion of females (18.7% vs. 10.1%, *p* = 0.133) than patients with non-cervical ESCC. Cervical ESCC had worse differentiation (34.4% vs. 19.6%, *p* = 0.049 in the rate of poorly differentiated carcinoma) and higher T stage (6.2% vs. 37.2%, *p* = 0.003 in T1; 28.1% vs. 4.7%, *p* < 0.001 in T4) than non-cervical ESCC. There was no difference in lymph node metastasis (65.6% vs. 54.6%, *p* = 0.229) or distant metastasis (15.6% vs. 15.5%, *p* = 0.983) between the two groups. Overall survival was lower in the cervical group (hazard ratio [HR], 1.51; 95% confidence interval [CI], 0.96–2.40; *p* = 0.076). When comparing outcomes of patients who underwent definitive chemoradiotherapy, the complete response rate (63.6% vs. 53.4%, *p* = 0.340) and survival (HR, 1.01; 95% CI, 0.51–1.99; *p* = 0.973) were similar between the two groups. **Conclusions:** Although cervical ESCC had a poor prognosis due to its higher T-stage and worse differentiation compared to other locations, outcomes were similar when treated with definitive chemoradiotherapy.

## 1. Introduction

Esophageal squamous cell carcinoma (ESCC) occurs frequently in older individuals, and its prevalence increases as life expectancy increases [1,2]. Squamous cell carcinoma (SCC) is the most common histological type of esophageal cancer, and it is overwhelmingly predominant in East Asia countries [2,3]. The cervical esophagus is the short part of the esophagus between the lower border of the cricoid cartilage and the suprasternal notch [4]. The majority of ESCCs are found in the mid-thoracic region, while cervical ESCCs account for only about 2–10% of ESCCs [5]. Cervical ESCC is treated nonoperatively, given the difficulty of re-anastomosing the esophagus close to the larynx. Therefore, definitive chemoradiotherapy (CRT) is the standard treatment modality recommended by guidelines [6,7]. Because surgical resection of cervical esophageal cancer is difficult, the prognosis is expected to be worse than that of esophageal cancers in other locations. However, there has been no study comparing it with other locations. Thus, through a single-center retrospective study, we aimed to analyze the clinical characteristics and prognosis of cervical ESCC in comparison with ESCC located in other regions.

## 2. Materials and Methods

### 2.1. Patients

Medical records of patients with esophageal cancer treated between December 2008 and December 2024 at Pusan National University Yangsan Hospital (South Korea) were reviewed. Among patients histologically diagnosed with SCC, those without confirmed staging or follow-up data were excluded. A total of 497 patients with ESCC were finally included in the analysis. These patients were divided into cervical and non-cervical groups. Cervical ESCC was defined as ESCC located 15 to 20 cm from the incisor teeth on endoscopy [8]. Patients were followed from the time of diagnosis until death, and surviving patients were followed up until December 2024.

This study was approved by the Ethics Committee of our center’s review board (Institutional Review Board no. 55-2025-019, ethical approval date 10 February 2025). The requirement for informed consent was waived by the Ethics Committee of our center’s review board because the participants’ medical records were anonymized before analysis. All methods were performed in accordance with the relevant guidelines and regulations by the Ethics Committee of Pusan National University Yangsan Hospital review board.

### 2.2. Staging Workup

All patients included in this study underwent upper gastrointestinal endoscopy, endoscopic ultrasound (EUS), chest/abdominal computed tomography (CT), and ^18^F-fluorodeoxyglucose (FDG) positron emission tomography/computed tomography (PET/CT) before treatment. T stage was measured through EUS, and invasion of adjacent structures was confirmed through EUS and CT findings. Lymph node metastasis was confirmed through EUS, CT, and FDG-PET/CT findings. Distant metastasis was confirmed by CT and FDG-PET/CT. ESCC was staged according to the American Joint Committee on Cancer staging system, revised 8th edition [9].

### 2.3. Surgery

Patients who received surgery underwent Ivor Lewis esophagectomy with an intrathoracic anastomosis and two-field or three-field lymph node dissection. Patients with anatomical abnormalities, such as those who have undergone gastrectomy, have undergone jejunal or colonic interposition. One patient with cervical ESCC received surgery underwent esophagectomy with laryngectomy. All patients underwent circular stapled anastomosis. Anastomotic leakage was assessed at one week after surgery using esophagography and endoscopy. A pathological examination was conducted to evaluate complete (R0) resection and the presence of lymph node metastasis.

### 2.4. Chemoradiotherapy

CRT was administered either with curative intent as definitive CRT or as neoadjuvant CRT prior to surgery. Based on individual clinical conditions, some patients received definitive radiotherapy (RT), while others underwent palliative RT for symptom relief. Radiation was delivered using either three-dimensional conformal radiotherapy or intensity-modulated radiotherapy. RT protocols were as follows: (1) definitive CRT, 50 Gy in 25 fractions (2 Gy per fraction); (2) neoadjuvant CRT, 45 Gy in 25 fractions (1.8 Gy per fraction); and (3) palliative RT, 30 Gy in 10 fractions (3 Gy per fraction). Chemotherapy regimens primarily consisted of either cisplatin and 5-fluorouracil (cisplatin 75 mg/m^2^ on day 1 and 5-fluorouracil 750 mg/m^2^/day for 4 consecutive days during weeks 1 and 5), or paclitaxel and carboplatin (paclitaxel 50 mg/m^2^ and carboplatin at an area under the curve of 2 mg/mL/min, administered weekly on day 1 for 5 weeks).

### 2.5. Histopathological Evaluation

All patients in this study were diagnosed with ESCC through endoscopic biopsy. The standard pathology protocol was to confirm the degree of cancer differentiation. However, there were some patients who only had biopsy results without a record for cancer differentiation from outside hospitals. For some patients, cancer differentiation was unclear. Surgically resected primary tumors and lymph nodes were sliced at 4-mm intervals. Endoscopically resected primary tumors were sliced at 2 mm intervals. Two pathologists with more than five years of experience independently assessed specimens of primary tumors and lymph nodes to determine tumor invasion depth, macroscopic type, differentiation, lympho-vascular invasion status, and nodal metastasis. All specimens were pathologically reviewed according to the Japanese Classification of Esophageal Cancer (11th edition) [10].

### 2.6. Follow-Up After Treatment

Following curative treatment—including surgery, definitive CRT, or endoscopic resection—patients underwent surveillance with endoscopy and chest/abdominal CT at 2–3 months post-treatment, every 3 months during the first year, and every 6 months thereafter. FDG-PET/CT was not routinely performed after surgery or endoscopic resection but was typically conducted 3–6 months after completion of definitive CRT. Surgical resection was generally performed within 2 months after completion of neoadjuvant CRT. However, in selected cases, patients were managed non-operatively based on preoperative evaluation results and overall clinical condition, as determined through a multidisciplinary team approach. Clinically complete response (cCR) after CRT was defined as no evidence of cancer in examinations performed 2–3 months after completing CRT, including endoscopy, endoscopic biopsy, chest/abdominal CT, and FDG-PET/CT. For ambiguous examination results due to esophagitis or ulcers following CRT, the examinations were repeated after 2–3 months.

### 2.7. Statistical Analysis

Student’s *t*-test and Fisher’s exact test were used to analyze categorical and continuous variables, respectively, for in-group comparisons. The Kaplan–Meier method was used to estimate survival, and the log-rank test was used to determine significant differences between groups. A Cox regression model was used to estimate the hazard ratio (HR) associated with survival. Statistical significance was set at *p* < 0.05. The Statistical Package for the Social Sciences version 27.0 (IBM Corp., Armonk, NY, USA) was used for statistical analyses.

## 3. Results

### 3.1. Baseline Characteristics of Patients

The median age of the 497 patients was 68 (range, 41–95) years. Of the patients, 89.3% (n = 444) were males. The median follow-up period was 20 months (range, 1–192 months). Regarding cancer location, 6.4% (n = 32) were in the cervical region, 15.1% (n = 75) in the upper thoracic region, 45.1% (n = 224) in the mid-thoracic region, and 33.4% (n = 166) in the lower thoracic region. Poorly differentiated carcinoma accounted for 20.5% (n = 102). Lymph node metastases were present in 55.3% (n = 275) of patients, and distant metastases were present at diagnosis in 15.5% (n = 77) of patients. Regarding main treatment, 46.1% (n = 229) of patients underwent surgery, and 20.7% (n = 103) of patients underwent CRT or RT. Baseline characteristics of patients are summarized in Table 1.

### 3.2. Cervical Esophageal Cancer

Results of comparing the cervical group (n = 32) and the non-cervical group (n = 465) are summarized in Table 2. The cervical group was older at the time of diagnosis (median, 73 [range, 46–87] years vs. 67 [range, 41–95] years; *p* = 0.047) and the proportion of women trended higher (18.7% vs. 10.1%, *p* = 0.133) compared to the non-cervical group. The cervical group had worse differentiation (34.4% vs. 19.6%, *p* = 0.049 in the rate of poorly differentiated carcinoma) and higher T stage (6.2% vs. 37.2%, *p* = 0.003 in T1; 28.1% vs. 4.7%, *p* < 0.001 in T4) than the non-cervical group. On the other hand, there was no significant difference in lymph node metastasis (65.6% vs. 54.6%, *p* = 0.229) or distant metastasis (15.6% vs. 15.5%, *p* = 0.983) between the two groups. Among patients in the cervical group, most patients (68.8%, n = 22) underwent definitive CRT or RT, while only one patient underwent surgery.

### 3.3. Definitive Chemoradiotherapy

A total of 95 patients underwent definitive CRT, including 22 in the cervical group and 73 in the non-cervical group. Age, sex, differentiation, stage, lymph node, and distant metastasis, and chemotherapy regimen showed no significant differences between the two groups. The proportions of patients who died during CRT were comparable between the cervical and non-cervical groups (4.5% vs. 2.7%, *p* = 0.674), as were the proportions of those who were unable to complete the planned CRT schedule due to adverse effects (9.1% vs. 13.7%, *p* = 0.571). The rate of achieving cCR after CRT did not differ significantly between the two groups (63.6% vs. 53.4%, *p* = 0.340). No statistically significant differences were observed according to disease stage. Among patients who achieved cCR, cancer recurrence occurred in two patients (14.3%) in the cervical group and five patients (12.8%) in the non-cervical group during the follow-up period. A comparison between the two groups of patients who underwent definitive CRT is summarized in Table 3.

### 3.4. Survival

**Figure 1:**
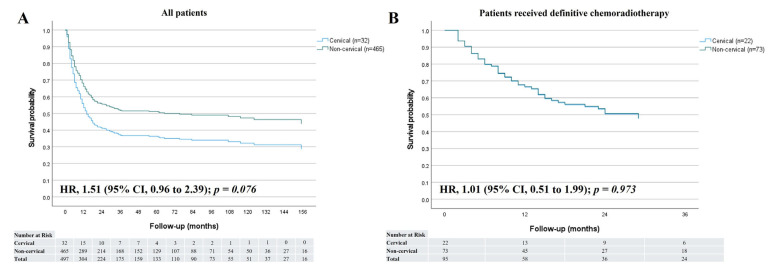
Overall survival curves for cervical and non-cervical groups. (**A**) Survival for the two groups of all patients. (**B**) Survival for the two groups of patients who received definitive chemoradiotherapy. HR, hazard ratio; CI, confidence interval.

**Figure 2:**
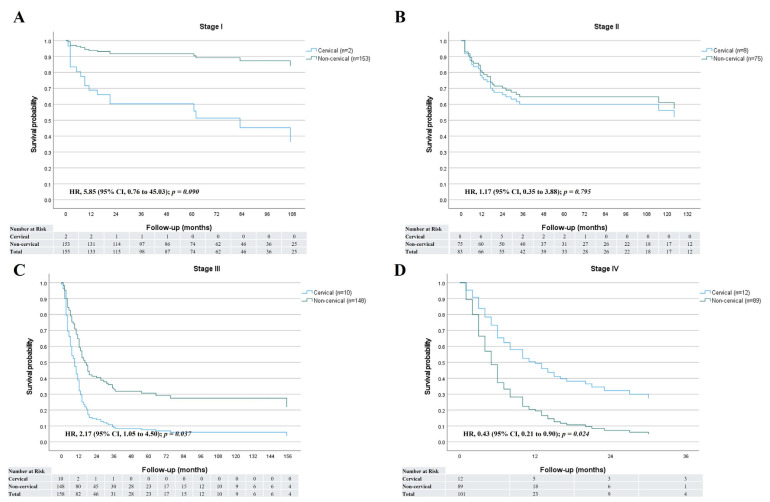
Comparison of overall survival curves between the two groups according to stages. (**A**) Stage I. (**B**) Stage II. (**C**) Stage III. (**D**) Stage IV. HR, hazard ratio; CI, confidence interval.

The median follow-up period was 10 months (range, 1–141 months) for the cervical group and 20 months (range, 1–192 months) for the non-cervical group. Of surviving patients (12 cervical and 241 non-cervical), the median follow-up duration was 38 months (range, 8–141 months) for the cervical group patients and 60 months (range, 5–192 months) for the non-cervical group patients. Overall, patient survival trended lower in the cervical group (HR = 1.51, 95% confidence interval [CI] 0.96–2.39, *p* = 0.076) (Figure 1A). However, survival after definitive CRT (HR = 1.01 [95% CI 0.51–1.99], *p* = 0.973) was similar between the two groups (Figure 1B). Overall survival rates at 1, 3, and 5 years were 49.2%, 35.8%, and 35.8%, respectively, in the cervical group, and 66.4%, 52.0%, and 51.4%, respectively, in the non-cervical group. Overall survival rates after definitive CRT or RT at 1, 3, and 5 years were 62.8%, 47.8%, and 47.8%, respectively, in the cervical group and 67.8%, 47.9%, and 47.9%, respectively, in the non-cervical group. When survival was compared by disease stage, the cervical group generally showed worse outcomes than the non-cervical group. However, among patients with stage IV disease, the cervical group demonstrated better survival (HR = 0.43; 95% CI, 0.21–0.90; *p* = 0.024) (Figure 2).

### 3.5. Representative Cases

**Figure 3:**
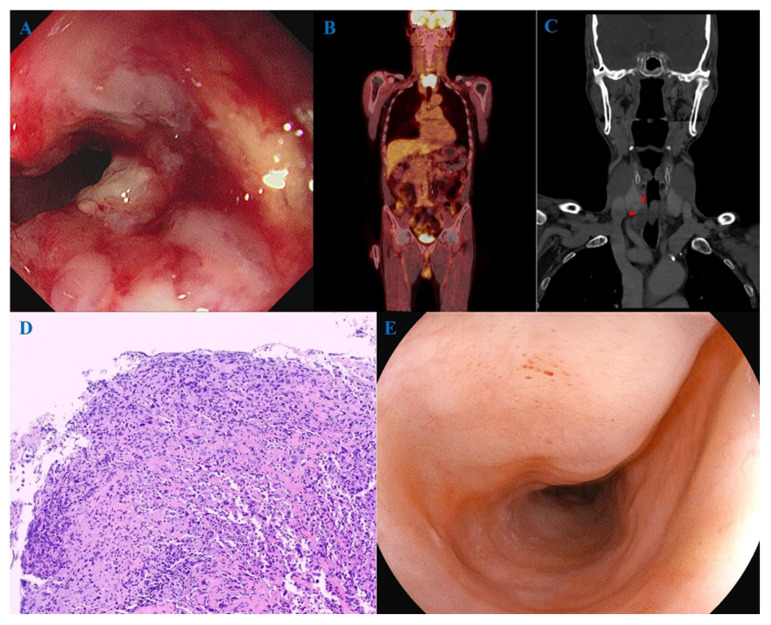
A 79-year-old male patient with cervical esophageal cancer. (**A**) An infiltrative mass in the cervical esophagus was observed on endoscopy. (**B**) FDG-PET/CT showed cervical esophageal cancer and surrounding lymph node metastasis. (**C**) Neck CT suspected direct invasion of the inferior aspect of the right thyroid gland (red arrows). (**D**) Endoscopic biopsy confirmed poorly differentiated carcinoma (hematoxylin and eosin, ×200). (**E**) Endoscopic findings 20 months after completion of definitive chemoradiotherapy showed no evidence of cancer. FDG-PET/CT, ^18^F-fluorodeoxyglucose-positron emission tomography/computed tomography.

The first case was a 79-year-old male patient with an infiltrative mass in the cervical esophagus observed on endoscopy (Figure 3A). FDG-PET/CT showed cervical esophageal cancer and surrounding lymph node metastasis (Figure 3B). Neck CT suspected direct invasion of the inferior aspect of the right thyroid gland (Figure 3C). Endoscopic biopsy confirmed poorly differentiated carcinoma (Figure 3D). The patient underwent definitive CRT with cisplatin/5-fluorouracil and achieved cCR. Endoscopic findings at 20 months after completion of CRT showed no evidence of cancer (Figure 3E).

**Figure 4:**
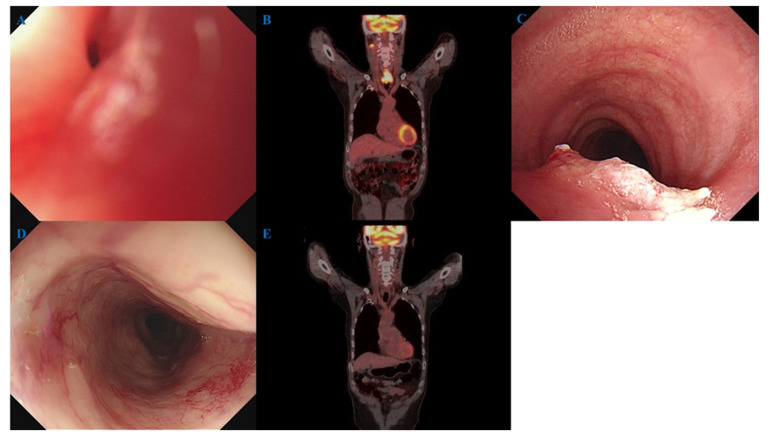
A 56-year-old male patient with cervical esophageal cancer. (**A**) The endoscope cannot be inserted due to a mass in the cervical esophagus. (**B**) FDG-PET/CT showed cervical esophageal cancer and lymph node metastasis in right neck level II. (**C**) Trachea invasion was observed on bronchoscopy. (**D**) Endoscopic findings 14 months after completion of definitive chemoradiotherapy showed no evidence of cancer. (**E**) FDG-PET/CT showed no evidence of cancer. FDG-PET/CT, ^18^F-fluorodeoxyglucose-positron emission tomography/computed tomography.

The second case was a 56-year-old male patient with obstruction due to a mass in the cervical esophagus (Figure 4A). FDG-PET/CT showed cervical esophageal cancer and lymph node metastasis in the right neck at level II (Figure 4B). Trachea invasion was observed on bronchoscopy (Figure 4C). The patient underwent definitive CRT with cisplatin/5-fluorouracil and achieved cCR. Fourteen months after completion of CRT, endoscopic findings (Figure 4D) and FDG-PET/CT (Figure 4E) showed no evidence of cancer.

## 4. Discussion

In the present study, cervical esophageal cancer had a higher proportion of T4 stage and poorly differentiated carcinoma compared to ESCC at other locations, and thus a worse prognosis. However, in patients with cervical esophageal cancer who underwent definitive CRT, cCR and prognosis did not differ from those with ESCC in other locations. Failure to complete the CRT schedule due to side effects or mortality during CRT was not different from that in other locations. The results of our study may serve as evidence for actively performing CRT for patients with cervical esophageal cancer even at advanced stages.

The reason cervical esophageal cancer is diagnosed in an advanced stage is that it is not detected early. Since the upper esophagus is where the endoscope is inserted, early-stage upper esophageal cancer may be missed during screening endoscopy, delaying its diagnosis [11]. In particular, the cervical esophagus is observed as a blind spot due to the upper esophageal sphincter during endoscope entry and withdrawal. In our study, older age among patients with cervical esophageal cancer also appeared to be associated with delayed diagnosis. The reason why the T4 stage is common in cervical esophageal cancer might be because there are many structures and organs in the narrow neck. Compared to other locations, there are many structures and organs in a narrow space of the cervical esophagus. Thus, even if cancer progresses only slightly, there is a high possibility that it will invade surrounding structures. Another study of cervical esophageal cancer using the Surveillance, Epidemiology and End Results database has also reported that 35% of cases have the T4 stage [4].

In our study, cervical esophageal cancer had a high proportion of poorly differentiated carcinoma. However, we could not find any evidence related to this finding. The location of the cervical esophagus is close to the head and neck. Head and neck squamous cell carcinoma (HNSCC) is highly related to human papillomavirus (HPV). HPV-positive HNSCC has a high proportion of poorly differentiated carcinoma [12]. In addition to tobacco and alcohol, some studies have determined the relationship between HPV infection and risk factors for ESCC [13,14,15]. One of our research results is that the proportion of women with cervical esophageal cancer is slightly higher than that in other locations. This might be due to other factors besides tobacco and alcohol. HPV-positive SCC is known to be more sensitive to radiation [16,17].

Many studies have reported the effectiveness of definitive CRT for cervical esophageal cancer. Definitive CRT is also suggested as a standard treatment in guidelines [6,7]. However, the radiation dose and chemotherapy regimen of definitive CRT for cervical esophageal cancer have not been established. Whether it should follow the treatment for ESCC or HNSCC has not been determined either [5]. The most evidence-based CRT regimen for cervical esophageal cancer is the combination of cisplatin and 5-fluorouracil [18,19,20]. The recommended total radiation dose for definitive CRT for ESCC is 50–50.4 Gy [6], whereas 66–70 Gy is recommended for HNSCC [21]. In our study, definitive CRT was performed for cervical esophageal cancer not differently to esophageal cancer in other locations. The most common chemotherapy regimen was a combination of cisplatin/5-fluorouracil, and the total radiation dose was 50 Gy. Results of definitive CRT have been reported in a meta-analysis of 1222 patients with cervical esophageal cancer, with a cCR rate of 58.6%, and estimated pooled overall survival rates at 1, 3, and 5 years of 77.9%, 48.4%, and 35.3%, respectively [22]. These are similar to our study results showing a cCR rate of 63.6% and overall survival rates at 1, 3, and 5 years after definitive CRT of 67.8%, 47.9%, and 47.9%, respectively. And in our study, overall survival rates after definitive CRT in the cervical group were not different from those in the non-cervical group. Moreover, in our study, patients with stage IV disease in the cervical group showed better survival compared to those in the non-cervical group, which may be attributed to the more aggressive application of definitive CRT in cervical ESCC patients, even at stage IV. However, given the retrospective nature of this study, selection bias in treatment decisions may have influenced the results. Additionally, the small sample size and reliance on *p* values alone limit the robustness of statistical interpretation.

This study had some limitations. This was a single-center retrospective study with a small number of patients, particularly those with cervical esophageal cancer. Due to the small sample size, multivariate analysis could not be performed. Second, there may have been bias due to the inclusion of patients who received definitive CRT in the non-cervical group who had poor condition and could not undergo surgery. Due to the limited number of patients and potential selection bias, some *p* values in this study may indicate a trend rather than a clinically significant difference. Third, although the staging was based on all test results, it may not have been accurate because all patients with cervical esophageal cancer had clinical staging except for one who underwent surgery. Fourth, while a potential association between HPV infection and cervical esophageal cancer was hypothesized, HPV-related testing was not performed at the time of diagnosis and therefore could not be retrospectively analyzed to provide supporting evidence. Fifth, the shorter follow-up duration in patients with cervical esophageal cancer may have led to an underestimation of their prognosis. In addition, treatment-related toxicity and post-treatment quality of life were not evaluated.

## 5. Conclusions

Cervical ESCC had a higher incidence of poorly differentiated carcinoma and T4 stage, with worse prognosis than ESCC in other locations. However, when definitive CRT was performed, results and prognosis were similar to those of ESCC in other locations. Further studies are needed to investigate a larger number of patients with cervical esophageal cancer and the relationship to HPV.

## Figures and Tables

**Table 1:** Baseline characteristics of patients.

Characteristics	
**Total, n**	497
**Age, median (range) years**	68 (41~95)
**Male, n (%)**	444 (89.3)
**Location, n (%)**	
*Cervical*	32 (6.4)
*Upper thoracic*	75 (15.1)
*Mid-thoracic*	224 (45.1)
*Lower thoracic*	166 (33.4)
**Differentiation, n (%)**	
*Well*	105 (21.1)
*Moderate*	198 (39.8)
*Poorly*	102 (20.5)
*Unknown*	92 (18.5)
**T stage, n (%)**	
*1*	175 (35.2)
*2*	56 (11.3)
*3*	235 (47.3)
*4*	31 (6.2)
**Lymph node metastasis, n (%)**	275 (55.3)
**Initially distant metastasis, n (%)**	77 (15.5)
**Stage, n (%)**	
*I*	155 (31.2)
*II*	83 (16.7)
*III*	158 (31.8)
*IV*	101 (20.3)
**Main treatment, n (%)**	
*Operation*	229 (46.1)
*Chemoradiotherapy or radiotherapy*	103 (20.7)
*Endoscopic resection*	57 (11.5)
*Chemotherapy*	38 (7.6)
*None*	70 (14.1)

**Table 2:** Comparison of cervical and non-cervical groups.

	Cervical (n = 32)	Non-Cervical (n = 465)	*p* Value
**Age, median (range) years**	73 (46~87)	67 (41~95)	0.047
**Male, n (%)**	26 (81.3)	418 (89.9)	0.133
**Differentiation, n (%)**			
*Well*	4 (12.5)	101 (21.7)	0.224
*Moderate*	12 (37.5)	186 (40.0)	0.887
*Poorly*	11 (34.4)	91 (19.6)	0.049
*Unknown*	5 (15.6)	87 (18.7)	0.664
**T stage, n (%)**			
*1*	2 (6.2)	173 (37.2)	0.003
*2*	7 (21.9)	49 (10.5)	0.056
*3*	14 (43.8)	221 (47.5)	0.679
*4*	9 (28.1)	22 (4.7)	<0.001
**Lymph node metastasis, n (%)**	21 (65.6)	254 (54.6)	0.229
**Initially distant metastasis, n (%)**	5 (15.6)	72 (15.5)	0.983
**Stage, n (%)**			
*I*	2 (6.2)	153 (32.9)	0.007
*II*	8 (25.0)	75 (16.1)	0.198
*III*	10 (31.3)	148 (31.8)	0.946
*IV*	12 (37.5)	89 (19.1)	0.015
**Main treatment, n (%)**			
*Operation*	1 (3.1)	228 (49.0)	0.001
*Chemoradiotherapy or radiotherapy*	22 (68.8)	81 (17.4)	<0.001
*Endoscopic resection*	0 (0)	57 (12.3)	0.122
*Chemotherapy*	2 (6.2)	36 (7.7)	0.759
*None*	7 (21.9)	63 (13.5)	0.196

**Table 3:** Comparison of two groups that underwent definitive chemoradiotherapy.

	Cervical (n = 22)	Non-Cervical (n = 73)	*p* Value
**Age, mean (range) years**	68 (46~87)	72 (53~89)	0.566
**Male, n (%)**	18 (81.8)	69 (94.5)	0.075
**Differentiation, n (%)**			
*Well*	4 (18.2)	13 (17.8)	0.887
*Moderate*	9 (40.9)	35 (47.9)	0.562
*Poorly*	6 (27.3)	10 (13.7)	0.143
*Unknown*	3 (13.6)	15 (20.5)	0.472
**T stage, n (%)**			
*1*	2 (9.1)	9 (12.3)	0.679
*2*	7 (31.8)	14 (19.2)	0.215
*3*	7 (31.8)	44 (60.3)	0.022
*4*	6 (27.3)	6 (8.2)	0.025
**Lymph node metastasis, n (%)**	12 (54.5)	52 (71.2)	0.148
**Initially distant metastasis, n (%)**	1 (4.5)	5 (6.8)	0.699
**Stage, n (%)**			
*I*	2 (9.1)	9 (12.3)	0.679
*II*	7 (31.8)	15 (20.5)	0.276
*III*	7 (31.8)	36 (49.3)	0.153
*IV*	6 (27.3)	13 (17.8)	0.334
**Concurrent chemoradiotherapy, n (%)**	18 (81.8)	61 (83.6)	0.848
***Chemotherapy regimen***			
*5-Fluorouracil + Cisplatin*	12/18 (66.7)	46/61 (75.4)	0.463
*Paclitaxel + Carboplatin*	3/18 (16.7)	12/61 (19.7)	0.775
*Others*	3/18 (16.7)	3/61 (4.9)	0.119
**Radiotherapy alone, n (%)**	4 (18.2)	12 (16.4)	0.848
**Schedule completed, n (%)**	20 (90.9)	63 (86.3)	0.571
**Death during treatment, n (%)**	1 (4.5)	2 (2.7)	0.674
**Clinically complete response, n (%)**	14 (63.6)	39 (53.4)	0.340
***According to Stage***			
*I*	1/2 (50.0)	9/9 (100)	0.112
*II*	6/7 (85.7)	12/15 (80.0)	0.747
*III*	2/7 (28.6)	12/36 (33.3)	0.806
*IV*	5/6 (83.3)	6/13 (46.2)	0.151
***recurrence after complete response***	2/14 (14.3)	5/39 (12.8)	0.890

## Data Availability

The data presented in this study are available on request from the corresponding author due to ethical reasons.

## Abbreviations

ESCC: esophageal squamous cell carcinoma; SCC: squamous cell carcinoma; CRT, chemoradiotherapy; EUS: endoscopic ultrasound; CT: computed tomography; FDG: ^18^F-fluorodeoxyglucose; PET/CT: positron emission tomography/computed tomography; RT: radiation therapy; cCR: clinically complete response; HR: hazard ratio; CI: confidence interval; HNSCC: head and neck squamous cell carcinoma; HPV: human papilloma virus.
